# Could CH_3_-M6P Be a Potential Dual-Functioning Candidate for Bone Regeneration?

**DOI:** 10.3390/biomedicines12122697

**Published:** 2024-11-26

**Authors:** Fidan Huseynova, Cătălina Ionescu, Frederic Cuisinier, Irada Huseynova, Alamdar Mammadov, Véronique Barragan-Montero

**Affiliations:** 1 LBN, Montpellier University, 34193 Montpellier, France; 2 Institute of Molecular Biology and Biotechnologies, Minstry of Science and Education of the Republic of Azerbaian, AZ1073 Baku, Azerbaijan; 3 Department of Cytology, Embryology and Histology, Azerbaijan Medical University, AZ1078 Baku, Azerbaijan; 4 Department of Chemistry, Faculty of Sciences, University of Craiova, 107i Calea București, 200144 Craiova, Romania; catalinagurgui@yahoo.co.uk; 5 Centre de Soins et de Recherche Dentaire, CHU Montpellier, 34000 Montpellier, France; 6 Faculté d’Odontologie, Montpellier University, 34295 Montpellier, France

**Keywords:** CH_3_-M6P, stem cell, DPSC, bone formation, angiogenesis, gene expression

## Abstract

**Background**: CI-RM6P has different binding sites with affinities for both M6P and IGF2, plays a role in the regulation of the TGF-β and IGF pathways that is important for controlling cell growth and differentiation. We hypothesize that previously synthesised derivative of M6P could be an alternative candidate for bone tissue regeneration in terms of higher binding affinity, stability in human serum, low cost and temporal delivery. **Methods**: CH_3_-M6P is synthesised based on previously described protocol; mesenchymal origin of isolated DPSCs was assessed by flow cytometry and AR staining prior to alkaline phosphatase (ALP) activity test, qPCR to evaluate differentiation specific marker expression, immunofluoresence, and SEM/EDS to evaluate organic and inorganic matrix formation; and rat aortic ring model to evaluate angiogenic effect of molecule. **Results**: CH_3_-M6P upregulated ALP activity, the expression of the *ALP*, *Col1*, *RunX2*, *Mef2C*, *TGFβ1*, *TGFβ1R*, *TGFβ2*, and *Smad3* genes under osteogenic conditions. The results of immunofluorescence and SEM/EDS studies did not show enhancing effect on matrix formation. As we observed, the induction effect of CH_3_-M6P on the expression of angiogenic genes such as *SMAD3* and *TGFβ1R*, even under osteogenic conditions, within the scope of research, we checked the angiogenic effect of the molecule and compared it to VEGF, showing that the CH_3_-M6P is really angiogenic. **Conclusions**: Our findings provide an important clue for the further exploration of the molecule, which can be necessary to enhance the capability of the commonly used osteomedium, possibly leading to the development of bone-forming drugs and has the potential to be a dual-functioning molecule for bone tissue engineering.

## 1. Introduction

Bone regeneration is one of the main focus areas in regenerative medicine. Treatment of bone defects due to trauma, periodontal disease, tumor resection, or severe infections remains a critical challenge due to limited effective therapies.

Dental implants are widely used in patients. Their surface is designed with an ability to integrate easily with the surrounding bone may be due to a histologically observed lack of negative tissue response. Pre-implantitis is a significant adverse effect related to the replacement of dental implants, which is similar to periodontitis, where bacterial biofilm accumulation around the implant causes the release of cytokines and inflammation, thus stimulating osteoclasts, which leads to the support of bone resorption due to calcium leakage to the blood and thus potential implant failure [1].

Bone quantity, quality, and soft tissue conditions around the implant are risk factors for early failure. Sinus and alveolar ridge augmentation using grafts such as hydroxyapatite (HA) or tricalcium phosphate (TCP) could be an alternative to autografts or xenografts [2].

Stem cells are another promising approach for tissue engineering and several types of mesenchymal stem cells have been shown to enhance bone repair and regeneration. Nevertheless, many strategies including various cell-based techniques, bioactive materials, and the application of various growth factors have been developed to overcome the infiltration of microvessels into the bone, but clinical trials have not yet been suited to these expectations. In large-sized bone engineering, vasculature penetration from the host tissue into the transplant is restricted to a depth of a few hundred micrometers, which leads to limited cell viability in the central region, causing non-healing of the defect. Thus, still, there is a clinical need for reliable and effective strategies.

To date, numerous approaches have been studied, but each of them has certain limitations. The recent literature advocates for the use of the following approaches: (1) growth factors (especially VEGF) are presented as best practice; however, its dose-dependent adverse effects should be considered, including that a reasonable dosage may also lead to tumor progression, severe vascular leakage, and hypotension [3,4], as well as the half circulation life of growth factors, purification problems, and the high expense of the product. (2) Next is an epigenetic approach; however, for unstable miRNAs, designing an appropriate miRNA delivery system for a specific tissue, a specific molecular target, is challenging [5]. (3) And, finally, the preparation of the right cocktail is another approach; however, choosing a medium with the right supplements to avoid non-specific cell lineage during the co-culture of cells is another challenge.

Another strategy that we focused on in the present study is the use of small chemicals, not only due to their low cost and easy control of their delivery, but also because they are molecules that help the target cells to internalize receptors, consequently, leading to the activation of signaling cascades. Using these molecules, there is a possibility of inducing a multitude of signaling pathways at their first natural steps, which will eventually be involved in all subsequent steps of the cascades for making the required growth factors by the cells. This can similarly modify the endogenous stem cell fate. It can be beneficial for reprogramming cells without genetic manipulation [6,7] and can contribute to rejuvenating strategies. In the present study, we evaluate the effects of a new derivative of M6P on the osteodifferentiation of dental pulp stem cells, as the M6P ligand is involved in many cellular processes including the TGF-β pathway. The mechanism of action of TGF-β1 on bone regeneration remains unclear. However, TGF-β is more abundant in the bones compared to other tissues. TGF-β is stored in an inactive form, released from the bone matrix, and is activated in the bone microenvironment [8,9]. TGF-β acts as a chemoattractant for osteogenic cells and enhances the migration of osteoprogenitor cells [10]. However, it can also inhibit osteogenesis by different mechanisms depending on the cell density, differentiation stage of cells, and concentration [11,12]. TGF-β represses the expression of osteocalcin through the action of HDAC 4 and 5, and also interacts with Smad3 at the Smad3/Runx2 complex within the Runx2-binding DNA sequence, leading to the inhibition of osteoblast maturation [13]. “It also inhibits osteogenesis of BMMSC, and thus attenuates in vivo bone regeneration: in low concentration, TGF-β1 activates Smad3, and upregulates BMP2 expression in BMMSCs by binding to the bone morphogenetic protein 2 (BMP2) promoter. High TGF-β1 doses, resulting in the repression of BMP2 expression and bone formation in mice increase tomoregulin-1 levels” [14].

The derivatives of M6P are bioisostere analogs of M6P and display higher affinity and stronger stability in human serum than M6P itself [15]. The angiogenic effects of M6P derivatives have been shown previously [16,17]. Natural M6P ligand binds to the M6P/IGF2 receptor [18]. This interaction affects the TGF-β pathway [19]. The addition of M6P to the culture medium can interfere with the binding of TGF-β1 to its receptor [18], thus affecting the activation of TGF-β1 [20]. Similarly, the TGF-β pathway’s ligands are also thought to have both pro- and anti-angiogenic properties. Low TGF-β levels contribute to the angiogenic switch by upregulating angiogenic factors and proteinases. In contrast, high TGF-β levels inhibited endothelial cell growth, stimulated smooth muscle cell differentiation, and recruited and promoted basement membrane reformation [21].

At the same time, as the receptor contains an IGF2 binding domain, M6P ligands regulate the extracellular concentration of IGF2 either by blocking its binding (by reciprocal modulation) to the receptor, or by increasing the binding affinity, leading to increasing or decreasing levels of IGF2, which is partially contradictory [22,23]. IGF2 plays an important role in bone regeneration [24], and its role in angiogenesis [25,26] has been well studied.

Considering that the investigation of small chemicals or their derivatives could be a better alternative, here, we first synthesized the derivative of M6P, as described previously, and checked its toxic effect on DPSC, alkaline phosphatase activity, and other osteogenic markers. Apart from classic markers, we evaluated many other angiogenic/osteogenic coupling markers after a wide literature search, which presume the altering of their expression. Then, we evaluated its effect on mineralization during osteodifferentiation and compared its angiogenic effect with the control and VEGF-treated group on an ex vivo rat aortic ring model.

We conclude that the new derivative, CH_3_-M6P, increases the DPSC differentiation to osteogenic lineage to some extent, shows angiogenic properties, and provides us a key clue for alternative therapeutic perspectives to continue further experiments in this domain. However, some experimental limitations exist, such as the evaluation of protein and mRNA expression in animal tissue models. Altogether, these findings propose a further exploration of the M6P analog, which could effectively solve the limitations in engineering strategies caused by the lack of angiogenesis, enhance the capability of the commonly used osteomedium, or possibly lead to the development of bone-forming drugs.

## 2. Materials and Methods

### 2.1. Chemicals

All commercially available reactants were purchased from Sigma-Aldrich (St. Louis, MO, USA), Carlo Erba (Emmendingen, Germany), or VWR (West Chester, PA, USA).

### 2.2. Synthesis of Methyl 6-O-phosphate-α-D-mannopyranoside (CH_3_-M6P)

An amount of 10 mL of CH_2_Cl_2_ and 56 mg of DMAP (0.6 mmol, 0.3 eq.) were added to a solution containing 400 mg of methyl α-D-mannopyranoside (2.05 mmol, 1 eq.) in 5 mL of pyridine. The mixture was poured into iced water before adding 260 µL of POCl_3_ (2.25 mmol, 1.1 eq.). The mixture was then warmed to room temperature. After 1 h of stirring, the solvents were removed under reduced pressure. The crude product was dissolved in AcOEt, extracted with water, and lyophilized. After the addition of MeOH, salts were filtered and evaporation under reduced pressure gave a white solid [16,27,28,29].

### 2.3. Cell Culture

Dental pulp cells were recovered from extracted third molars of healthy patients after obtaining written informed consent. This protocol was approved by the local ethical committee (Comité de Protection des Personnes, Montpellier Hospital, France). In order to remove pulp tissue, the tooth was divided into two pieces under the weak flow of 2% chlorhexidine (600079, Pierre Fabre, Hong Kong) from a certain point. The soft tissue was removed carefully and was digested using 3 mg/mL of collagenase type I (Cas. No. 9001-12-1, Gibco, Waltham, MA USA) and 4 mg/mL of dispase (Cat. No. 17105-041, Gibco) solution for 1 h at 37 °C, followed by filtration through 70 μm Falcon strainers and incubation in αMEM supplemented with 10% fetal bovine serum (FBS), 100 U/mL penicillin, and 100 μg/mL streptomycin. Non-adherent cells were removed after 24 h by washing, and cells were incubated for 1 week at 37 °C, 5% CO_2_.

### 2.4. Flow Cytometric Surface Marker Expression Analysis

In order to verify the mesenchymal origin, flow cytometry was used to characterize the surface marker expression of cells. For this, the cells were detached with trypsin and the reaction was stopped by adding a culture medium. After, centrifugation cells were resuspended in FACS buffer and 2%PFA and incubated for 30 min at RT. After a second washing using FACS buffer, the cells were incubated at 4 °C for 1 h with 5 µL of each CD105, CD90, CD73, Stro1, and CD117 antibodies.

### 2.5. Osteodifferentiation

DPSC was cultured at P3-P5 using basal medium (αMEM supplemented with 10%SVF and 1% penistrepto) until enough confluency and was then differentiated. At 100% confluency, cells were treated with basal medium (BM) and osteomedium (OM, which is supplemented with glycerophosphate, l-ascorbate acid, and 10nM dexamethasone) over 14 and 21 days [30,31].

### 2.6. Cell Viability Assay

In order to assess cell viability and proliferation, MTT (3-(4,5-dimethylthiazol-2-yl)-2,5-diphenyltetrazolium bromide) assay was performed at 16 and 65 h. This colorimetric test is based on the reduction in a yellow tetrazolium salt (MTT) to purple formazan crystals by NAD(P)H-dependent oxidoreductase enzymes, which exist in metabolically active cells. In our experiments, 10,000 cells were seeded per well of a 96-well plate being pentaplicate. An amount of 100 µL of MTT solution was used for each well at 1 mg/mL concentration and 100 µL of isopropanol was used to solve formazan crystals [32]. A microplate reader was used to find spectrophotometric absorbance at 540 nm wavelength (ELX 800, BioTek, Winooski, VT, USA).

### 2.7. Alkaline Phosphatase Activity

In vitro osteogenic differentiation of DPSC was induced by supplementing the growth medium with 100 μM CH_3_-M6P. Changes in alkaline phosphatase (ALP) enzyme activity were measured by the ALP assay following 7 and 14 days of osteodifferentiation. For this, the cells were rinsed with PBS 3 times and treated with 300 μL of 4-nitrophenyl phosphate disodium salt solution (P-nitrophenyl Phosphate Liquid Substrate System, Sigma-Aldrich, Pcode: 1002610371, 263.05 g/mol) for 35 min at RT, and measured by spectrophotometer at 405 nm wavelength 3 times (the experiment was carried out in triplicate, at least). ALP activity assays were performed according to the manufacturer’s protocol

### 2.8. SEM/EDS

After 21 days of differentiation, the micro-composition of minerals created by cells was observed and evaluated by SEM and EDS. For this analysis, samples were fixed with 2.5% glutaraldehyde, rinsed in PBS dehydrated with ethanol in increasing concentrations, and then chemically dried with HDMS. Mineral content and microstructure of the differentiated samples were assessed in low vacuum (without metallization), with the microscope Quanta 200 FEG from FEI and the EDS system is the Ultim Max detector from Oxford Instrument (Abingdon, UK).

### 2.9. Immunofluorescence

Immunostaining was performed for type I collagen expression at 3 weeks of osteodifferentiation to evaluate organic matrix formation. The cells were fixed, permeabilized, blocked, and incubated with primary antibodies. Collagen I antibody (ab34710; Abcam, Cambridge, UK) was diluted at 1:80 and was incubated overnight at 4 °C. The cultures were incubated with a secondary anti-rabbit Alexa 488 antibody (ab150073) diluted at 1:150 and incubated for 2 h at room temperature. The washed cells were stained with 4′,6-diamidino-2-phenylindole (DAPI). Analyses were performed using a fluorescence microscope. The result was expressed as the intensity of collagen normalized by DAPI.

### 2.10. mRNA Quantification

Quantitative real-time reverse transcription–polymerase chain reaction (qRT-PCR) analysis was used to compare the relative expression of osteogenic and osteogenic/angiogenic coupling genes under different culturing conditions. Total RNA was extracted from DPSC at 1 or 2 weeks of osteodifferentiation using Machery Nager reagent (REF 740,955.50) according to the manufacturing instructions. The concentration of RNA was assessed by nanodrop, and the quality of RNA was measured by a bioanalyzer. After the satisfactory RIN values (with a minimum of 8.6 and most of them a maximum of 10) in the next setup, cDNAs were synthesized from samples with a relative absorbance ratio of 1.86 and 2.11 at 260/280, using RevertAid First Strand cDNA Synthesis Kit (K1622) from Thermo Scientific (Waltham, MA, USA). QRT-PCR was performed on a LightCycler 480 SW 1.5. (Applied Biosystems, Waltham, MA, USA) for the genes shown in Table 1. Some gene-specific primers were chosen from the literature and checked by USCS software (https://its.ucsc.edu/software/student-software.html, accessed on 23 October 2024), and the others were designed using the “NCBI primer design” software (https://www.ncbi.nlm.nih.gov/tools/primer-blast/, accessed on 23 October 2024) according to the sequences from the “Emsemble.org” database. Analyses were normalized to the GAPDH housekeeping gene. Relative expression was evaluated using the comparative CT method (2^−ΔCt^).

### 2.11. Rat Aortic Ring Model

The angiogenic potential of CH_3_-M6P was evaluated and compared with VEGF on the rat aortic ring model. Aortas from 3 OFA rats (8–10 weeks) were dissected, cross-sectioned (1 mm), and embedded in collagen (1 mg/mL), and then treated with 100 µM CH_3_-M6P and 30 ng/mL of VEGF, for the following days. The evaluation was performed by counting under an optic microscope on day 3, day 6, day 8, and day 10. The focus was frequently adjusted in order to accurately score all vessels growing in 3D culture. Microvessels were distinguished from fibroblasts on the basis of their greater thickness and cohesive pattern of growth. All branches were counted as separate vessels. An experiment was carried out with pentaplicate for each of the three rats. (Note: during counting rings that never sprouted were removed to avoid artificial results.) Additionally, in order to assess the presence of vessels, the rings were stained with fluorescently conjugated lectin against endothelial cells and observed by immunofluorescence microscopy.

### 2.12. Statistical Analysis

Data were analyzed by GraphPad Prism 7 (Grap Software, Inc., La Jolla, CA, USA). Each experiment was repeated at least three times. Depending on the Shapiro–Wilk test result, statistical analysis was performed by using a non-parametric or parametric *t*-test, or a one-way ANOVA. * *p* < 0.05 was considered significant.

## 3. Results

### 3.1. Characterization of Synthesized CH_3_-M6P

M6P has been obtained in 75% yield and the structure of the product has been confirmed by NMR (^1^H and ^13^C) and by mass spectrometry and suits the data from the literature [16,27,28]. The chemical shifts and coupling constants in the ^1^H NMR spectrum indicate the presence of the protons of the carbohydrate cycle, among which the presence of H_4_ is characteristic. It is usual that, in mannose series, H_4_ is coupled with H_3_ and H_5_ with equal coupling constants, giving a triplet signal (in this case, at 3.45 ppm, ^3^J_H4-H3_ (a,a) = ^3^J_H4-H5_ (a,a) = 9.4 Hz). The signal of the methyl group in the anomeric position is also identified, at 3.32 ppm, as a singlet, integrating three protons. Analysis of the ^13^C NMR spectrum indicates the presence of the seven carbon atoms in M6-P, with chemical shifts ranging from 55.0 to 101.1 ppm. Mass spectra allow for the identification of the product in both positive and negative ionization modes, as adducts with H or Na ions, at *m*/*z* = 275.4 [M+H]^+^ and 397.1 [M+Na]^+^, or at *m*/*z* = 273.2 [M-H]^−^, respectively.

^1^H NMR (400.13 MHz, CDO3D) δ (ppm): 3.32 (s, 3H); 3.32–3.36 (m, 1H); 3.45 (t, 1H, J = 9.4 Hz); 3.65 (dd, 1H, J = 3.4 Hz, J = 9.5 Hz); 3.82 (dd, 1H, J = 1.7 Hz, J = 3.4 Hz); 4.14–4.36 (m, 2H); 6.10 (d, 1H, J = 1.5 Hz).

^13^C NMR (100.62 MHz, CDO_3_D) δ (ppm): 55.0, 66.9, 69.8, 70.1, 70.6, 71.5, 101.1.

^31^P NMR (162 MHz, CDO_3_D) δ (ppm): 4.38.

MS (ESI/MeOH) *m*/*z*: 275.4 [M+H]^+^, 397.1 [M+Na]^+^, 273.2 [M-H]^−^.

Rf: 0.06 (2-propanol/Ammonia 4/6).

**Scheme 1:**
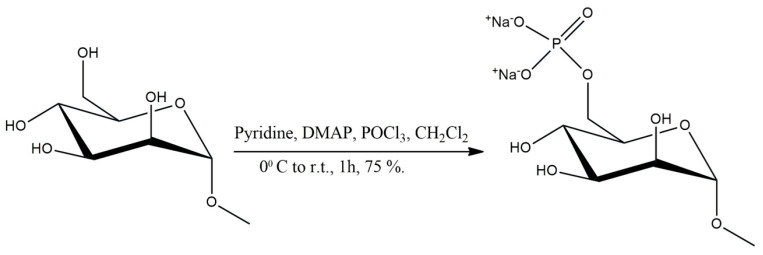
Synthesis of CH3-M6P. Reaction conditions and yield: pyridine, DMAP, POCl_3_, CH_2_Cl_2_, 0 °C to r.t., 1 h, 75%.

Yield: 75% (Scheme 1).

### 3.2. Flow Cytometry

**Figure 1:**
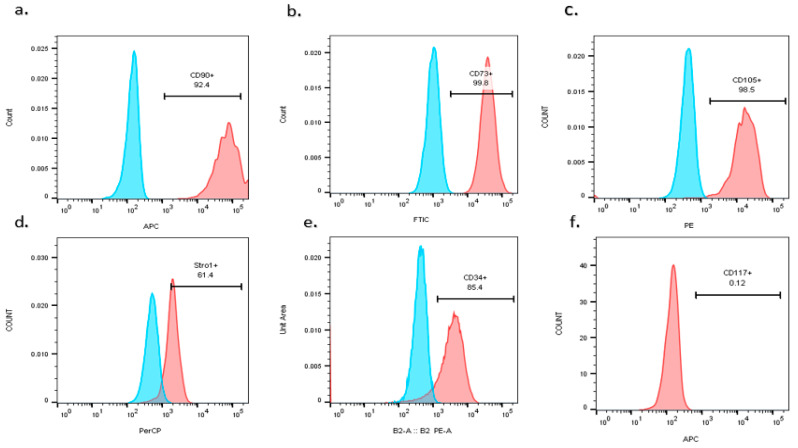
Identification of MSC markers by flow cytometry expressed on dental pulp cells. (**a**) CD90 is positive at 92.4%, (**b**) CD73 is positive at 99.8%, (**c**) CD105 is positive at 96.5%, (**d**) Stro1 is positive at 64.5% (**e**) CD34 is positive at 85.4%, (**f**) CD117 is negative. Blue spectra shows autoimmunofluoresence, while red spectra shows immunfluoresence of labeled antibody against marker.

In order to verify the mesenchymal origin, flow cytometry analyses were performed. The isolated pulp cells were positive for CD 105, CD73, CD90, and Stro1, but, at the same time, positive for CD34 and negative for CD117 (Figure 1).

CD105 (endoglin) is a component of the transforming growth factor-beta (TGFβ) receptor complex. CD73 acts as a cell adhesion molecule and mediates lymphocyte binding to endothelial cells. CD90 (Thy-1) is mainly present in leukocytes and is involved in cell–cell and cell–matrix interactions. All of these three markers have been reported to be expressed on DPSCs. STRO-1 is a mesenchymal stem cell marker and is also present in pericytes, some odontoblasts, and primary dental pulp tissue. CD34 and CD117 are commonly used to identify the hematopoietic stem cell population, but their expression by DPSC was reported [33].

### 3.3. Osteodifferentiation

**Figure 2:**
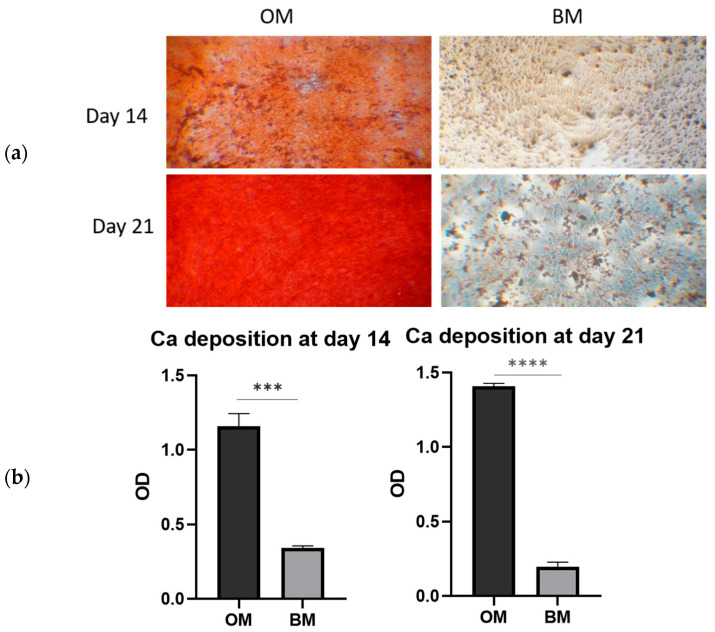
AR staining and assesment of Ca deposition. (**a**) Pictures obtained by optical microscopy at 14 and 21 days. The cells cultured with OM stained with red due to high amount of calcium deposition. (**b**) Graphs showing calcium deposition at 14 (*** *p* = 0.0007) and 21 days (**** *p* < 0.0001), assessed by spectrophotometric measurement (OM—osteomedium, BM—basal medium).

In order to prove the osteogenic differentiation ability of isolated DPSC, first of all, AR staining was performed using BM and classic OM. The obtained data showed that the cells are able to differentiate into osteoblastic lineage. The experiment was conducted in triplicate (Figure 2).

### 3.4. Cell Viability Test

**Figure 3:**
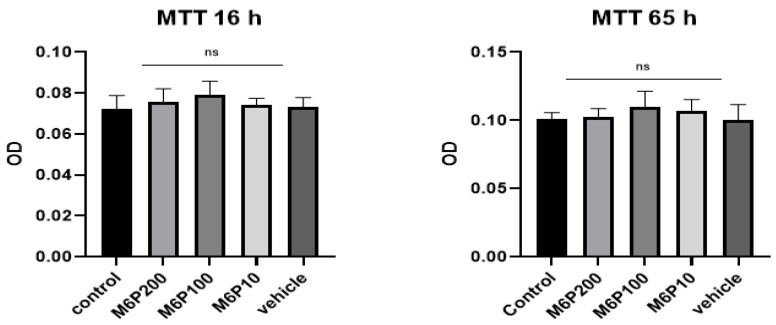
The effect of 200, 100, and 10 µM of methyl M6P on cell viability 16 h and 65 h after (*n* = 1, pentaplicat, ns—shows non-significant). (Vehicle is the mix of DMSO and water that was used to solve molecule).

An MTT assay was performed to check the molecule’s effect on DPSC viability. At 65 h of culture, 10, 100, and 200 µM of the tested compound was checked and revealed that analog has no effect on cell viability (Figure 3). The data were normally distributed. Comparisons were conducted by one-way ANOVA. There is no significant difference between cases.

### 3.5. CH_3_-M6P Effect on Osteodifferentiation

**Figure 4:**
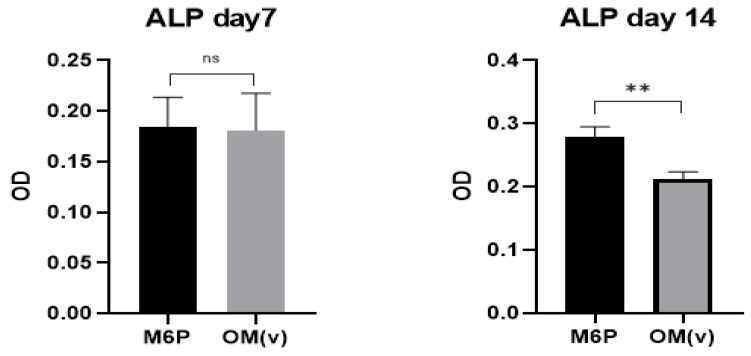
The effect of 100 µM of methyl M6P on alkaline phosphatase activity (ALP) at day 7 (*p* = 0.13, ns—shows non-significant) and day 14 (** *p* = 0.007) as compared to the control group (OM(v)—osteomedium with the vehicle) (*n* = 2, triplicate).

ALP is a ubiquitous protein, has an influential role in osteogenesis, and is a biological marker for bone turnover. An ALP assay was performed and the obtained data revealed that the methyl-M6P-treated group increased significantly at day 14 (*p* < 0.05) due to alkaline phosphatase activity, as compared to the control group (OM(v)) (Figure 4).

### 3.6. Inorganic Matrix Formation: SEM/EDS Spectroscopy

The mineralized phase of bone is composed of hydroxyapatite (HA) [Ca_10_(PO4)_6_(OH)_2_]. It gives strength and increases the mechanical resistance of the organic matrix. The amount of Ca in HA is 39.9%, of P is 18.5%, and the Ca/P ratio is quite variable in vertebrates but, in bone, the average corresponds to 1.67–2.16. But, because the biological environment consists of some collagen, non-collagen proteins, water, etc., the real ratio does not correspond to this value [34,35]. Thus, we decided to evaluate this value as one of the indicators to see the M6P-analog effect on this process. For this reason, SEM/EDS was performed after 3 weeks of osteodifferentiation.

**Figure 5:**
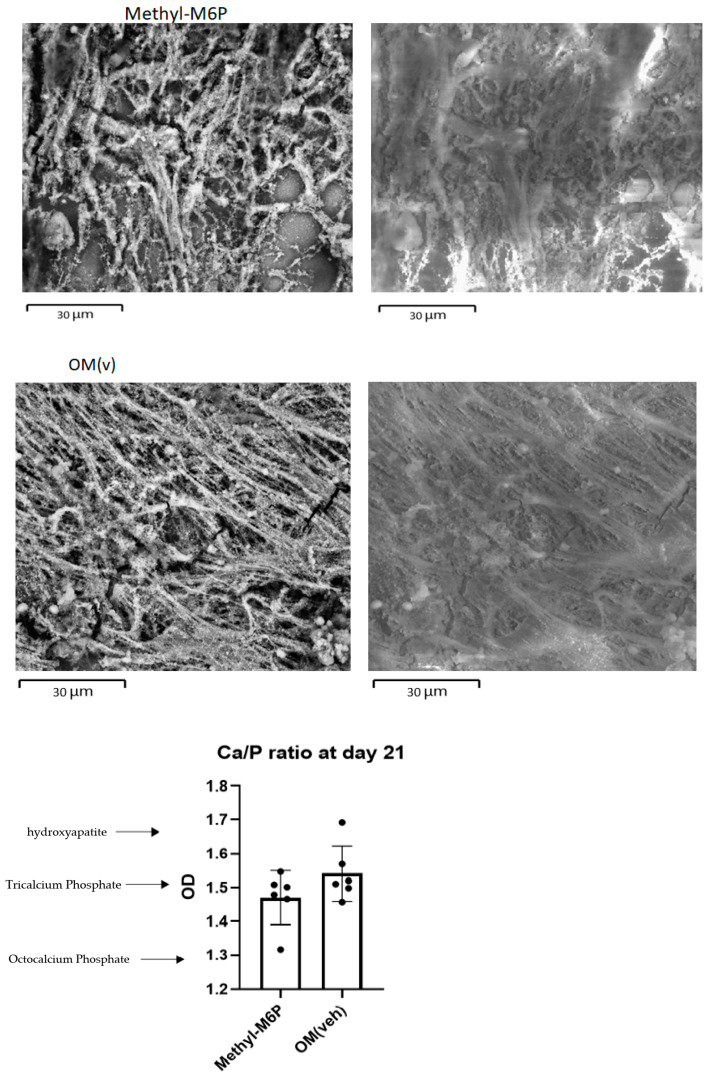
Picture obtained by SEM at 3 weeks of osteodifferentiation after treatment with methyl M6P and OM(v). The result in the graph was obtained by EDS and shows the Ca/P ratio for the same groups.

For the methyl-M6P-treated group, the micro-composition of minerals with 1.5 +/− 0.014 SEM suits Tricalcium cristal, however, with some areas close to octacalcium cristal, which is considered a mineral precursor to bone apatite crystals. For OM control, it was between tricalcium and hydroxyapatite with 1.55 +/− 0.0225 (Figure 5).

Tricalcium phosphate (TCP) is one of the most used constituents of bone substitutes, as well as hydroxyapatite. TCP exhibits lower mechanical strength than hydroxyapatite. This mineral is not just osteoconductive but also osteoinductive [36]. ß-TCP promoted hESCs differentiation, especially neural crest stem cell-related gene expressions and osteoblastic differentiation [37,38].

### 3.7. CH_3_-M6P Effect on Organic Matrix Formation

**Figure 6:**
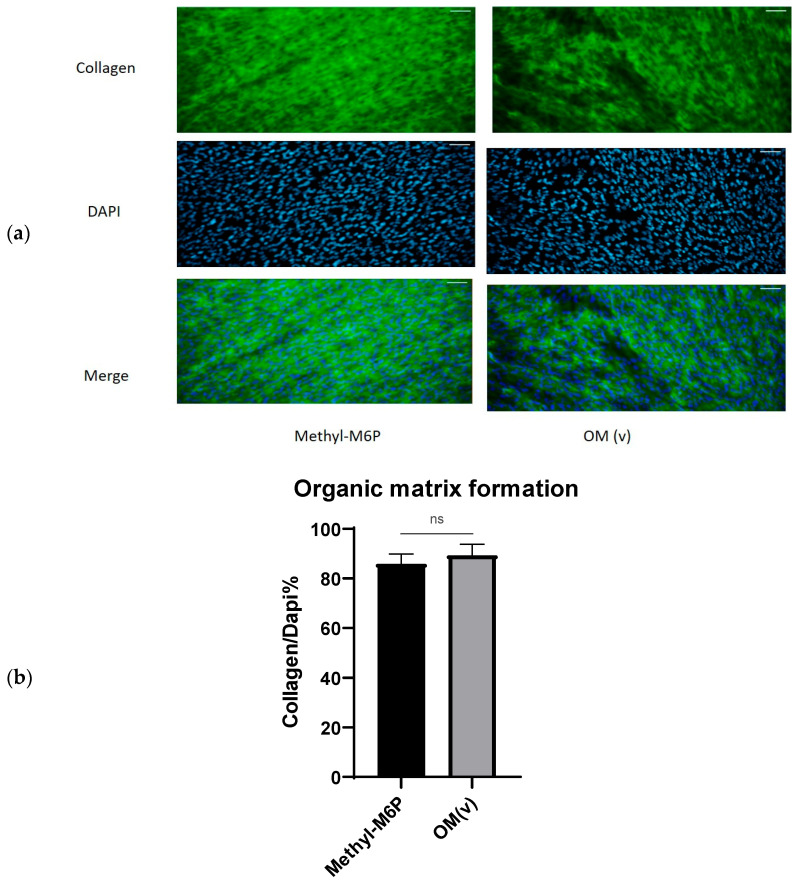
Top: (**a**) pictures obtained by immunofluorescence microscopy after immunostaining against collagen (scale bar is correspond to 83 μm). Bottom: (**b**) graph showing organic matrix formation at day 21 (tetraplicate). ns shows non-significant.

Bone tissue is characterized by its organic matrix as well as an inorganic matrix. The organic matrix of bone is composed mostly of collagen (90%). It is a scaffold for the cells and a reservoir for growth factors and other needed molecules. Thus we checked organic matrix formation, which is evaluated by the intensity of collagen/normalized by DAPI. The result of 3 weeks of culture showed that there is no significant difference in organic matrix formation after CH_3_-M6P treatment (*t*-test, *p* = 0.05) (Figure 6).

### 3.8. mRNA Quantification

**Figure 7:**
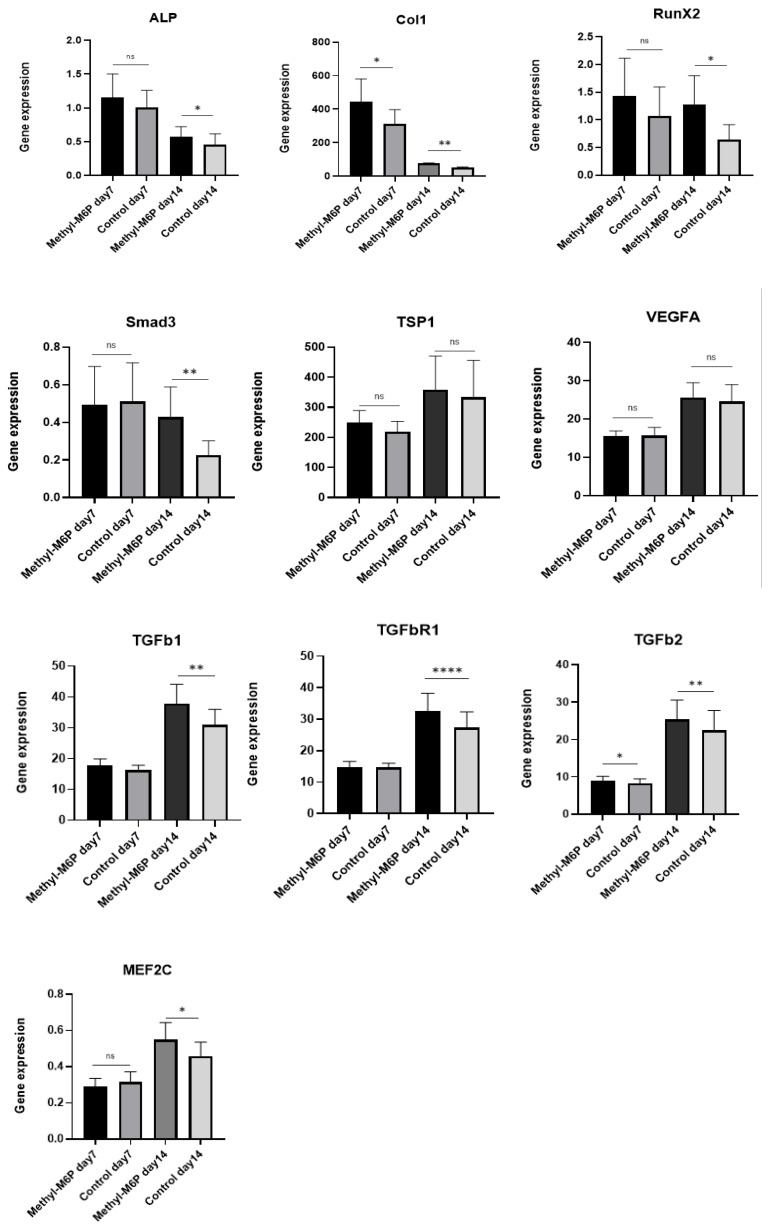
CH_3_-M6P effect on osteodifferentiation. The graphs show Col1, ALP, RunX2, Smad3, TSP1, VEGFA, TGFb1, TGFb1R, TGFb2, and Mef2C mRNA expression during 1 or 2 weeks of osteodifferentiation (*n* = 3, triplicate, ns = not significant, * *p* ≤ 0.05, ** *p* ≤ 0.01, **** *p* ≤ 0.0001).

Some osteogenic or angiogenic/osteogenic coupling genes were evaluated by predicting RT-PCR and may change after M6P treatment. The obtained absolute quantification (2^−ΔCt^ value) (Figure 7) revealed not high but significant differences for Col1, Mef2C, RunX2, ALP, Smad3, TGFβ1, TGFβ2, TGFβ1-R expression, but not for TSP-1, VEGFA expression. The experiment was conducted three times, in triplicate. A parametric or non-parametric *t*-test was performed depending on the Shapiro–Wilk test result.

### 3.9. Rat Aortic Ring Assay

**Figure 8:**
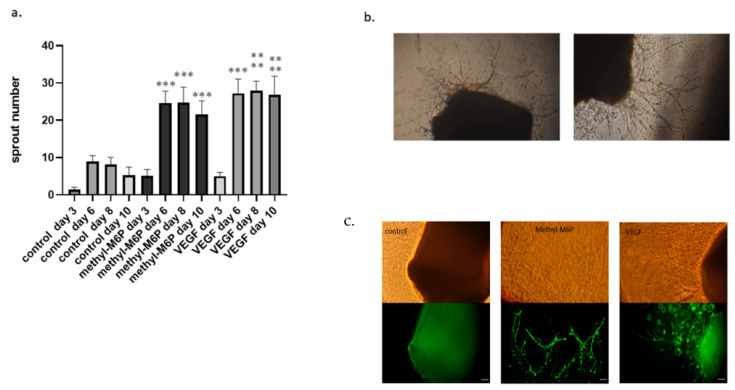
(**a**) Comparison of the angiogenic effect of 100 µM methyl M6P and 30 ng/mL VEGF on rat aortic ring model. *** *p* ≤ 0.001 and **** *p* ≤ 0.0001 were considered significant; * always shows the comparison against control at the same time point. (**b**) Assessing the existence of microvessels by immunostaining against lectin at the end of experiment (scale bar 100 µm). (**c**) Left picture, after treatment with 100 µM Methyl-M6P, and right picture, after treatment with 30 ng/mL VEGF, were obtained by light microscope on day 8 of living culture. (*n* = 3, pentaplicate).

The rat aortic assay was performed and the sprout was counted during the exponential growth phase. However, sometimes each ring and animal has an individual reaction, but data show that M6P has a better angiogenic effect than the control. Results from three rats show that the reaction against M6P started their degradation from day 8; however, VEGF started their degradation a little bit later than CH_3_-M6P (Figure 8).

## 4. Discussion

Since the lack of angiogenesis is the most prominent problem in bone regeneration, many researchers in this field try to overcome this problem.

Among a number of MSCs, DPSC seems to be the most promising approach for tissue engineering, both in terms of achieving them with simple in vitro protocols and its multipotency, which can differentiate multiple cell lineages including, dental tissue, nerves, and bone. They were isolated from third molars and exhibited a high frequency of colony formation that produced calcified nodules. Also, they have high angiogenic ability; however, DPSC lysates and their secretomes contain a wide range of anti-angiogenic factors as well as angiogenic factors. Despite the extensive literature with significant satisfactory results, clinical trials have not matched the expectations, due to a lack of vascularized stem cell scaffolds [39,40,41].

In our study, we could manage the osteogenic differentiation of DPSC in a positive way by the application of our previously adapted chemical.

After successful isolation, DPSCs were characterized based on their immunophenotyping for the surface markers using flow cytometry, and their osteogenic character was assessed by AR staining. CH_3_-M6P did not show a toxic effect on DPSC. CH_3_-M6P enhanced osteodifferentiation at 2 weeks of culture by inducing both ALP activity and ALP mRNA level. Also, the most prominent organic matrix component collagen 1 expression was about 30–50% induced in mRNA levels after one and two weeks of treatment; however, we did not observe a significant difference in assessing organic matrix formation at three weeks of treatment.

Upregulation of another master switch marker of osteodifferentiation RunX2, and angio/osteo coupling gene Mef2C after the treatment proves the osteogenic effect CH_3_-M6P, and, further, will be evaluated on the protein level. It was reported that Mef2C has an important role in cardiovascular and muscular mechanisms and bone development [42,43]. Mef2C has a role both in angiogenesis and osteogenesis, and deletion of this gene impairs cartilage angiogenesis, hypertrophy, ossification, and longitudinal bone growth in mice [42]. *Mef2c* gene silencing impairs osteodifferentiation and decreases ALP activity, mineralization, and knockdown expression of osteodifferentiation-specific genes such as osteocalcin and bone sialoprotein [42]. Also, the possible role of Mef2C in VEGF-dependent angiogenesis was reported. During targeted deletion of the gene, endothelial cells failed to organize in the normally vascular plexus [44].

There were slight but no significant differences in *VEGF* and *TSP1* expression, which also, in accordance with the literature, the expression of these two molecules in normal physiological conditions is always well balanced.

VEGF-A is a pro-angiogenic factor; parallelly, it plays a key role in the early stages of osteogenesis and has considerable functions in mechanisms underlying skeletal growth and repair [23]. In Human Mesenchymal Stem Cells, VEGF-A induces mineralization as an autocrine factor and the sprouting of endothelial cells as a paracrine factor [45].

TSP-1 is an anti-angiogenic factor, secreted by osteoblasts in developing skeleton, long, and calvarial bones and it is one of the main constituents of bone extracellular matrix proteins. *TSP-1* is significantly (10- to 15-fold) upregulated during the early phase of osteogenic differentiation of MC3T3-E1; however, it inhibits terminal osteoblast differentiation, and this action is completely parallel to ALP activity in the early stage. Due to this feature, TSP-1 has also been suggested to be considered an early marker of osteogenic differentiation [46]. The *TSP-1* promotor is a transcriptional target sequence of Runx2 [47]. However, it is also demonstrated that TSP-1 inhibits osteodifferentiation (indicated by decreased Runx2 and ALP expression) of human MSCs using latent TGF-β activation [48].

The expression of *TGFβ1*, *TGFβ1-R*, and *TGFβ2* at the mRNA level is not high but significantly induced after treatment. The increasing level of TGFβ and its receptors increases the sensitivity of the TGFβ pathway. We already mentioned that the role of TGF-β is, in part, contradictory, suggesting that in vitro results may not be strongly predictive of in vivo results. But, in all cases, TGF-β is abundant in bones compared with other tissues. TGF-β is stored in an inactive form here, released from the bone matrix, and activated in the bone microenvironment [8,9].

The increased expression level of angiogenic *Smad3* during osteodifferentiation also encourages us to think about further investigation of CH_3_-M6P’s role in regeneration. It was shown that Smad3 null mice have decreased bone density with a lower rate of bone formation, a reduced rate of osteoid width, and mineral deposition. As a critical component of the TGF-β pathway, it mimicked the effects of TGF-β—inhibited proliferation [9,49]—but it also enhanced ALP activity, mineralization, and the levels of bone matrix proteins such as type I collagen (COLI), OPN, and MGP on MC3T3-E1 cells [9]. At the same time, corresponding to one week of culture at the confluent state of MC3T3-E1 cells, Smad3 decreased Runx2 and OCN mRNA levels; however, at 14 and 21 days of cultures, the overexpression of Smad3 enhanced the mRNA level of Runx2 and OCN, showing that the expression of this gene depends on cell confluency and differentiation stage. It enhanced COLI and ALP mRNA, decreased expression of MEPE mineralization inhibitor at 7 days of cultures, and its effect on COL1 was decreased as the culture periods progressed. At the same time, its decreasing effect on the osteoblastic commitment of pluripotent cells indicates different effects of molecules depending on cell type [50]. However, its effect is ambiguous but it is a well-known angiogenic molecule, and angiogenesis is one of the main aspects of regeneration. Thus, it would be better to check the CH_3_-M6P effect in vivo, as in vivo involves both angiogenic and osteogenic processes. We again showed the high angiogenic effect of the M6P derivative on the rat aortic ring model and, additionally, compared it with VEGF. Its effect is lower compared to VEGF but the difference is not significant.

Coming to inorganic matrix formation, the micro-composition of the CH3-M6P-treated group not significantly but suits to tricalcium phosphate (TCP) and, in some areas, octacalcium phosphate (OCP). HA, TCP, and OCP are widely used as graft material. TCP is more soluble than HA, and thus relatively easily replaced by natural bone tissue due to higher porosity creating a better space for vessel penetration. OCP is a precursor to HA, osteoconductive, and degrades gradually by replacing new bone in the desired region, thus promoting better integration [51]. Biomimetic HA is a highly effective material for bone tissue engineering, including the dental and orthopedic sectors [52], in terms of osteoinductivity and long-term stability. As a complex nanocomposite of the mineral phase along with the organic matrix, bone tissue is organized into a porous structure [53] in which the porous nature of HA allows for blood vessels to penetrate through the material. To improve the usability of biomimetic HA, it is often combined with other components such as gel [52]. Hyaluronic gel integrates well with bone tissue, allowing its gradual replacement by natural tissue without immune rejection. It has the ability to interact with stem cells, enhance cell migration and proliferation, and create a hydrated environment, which is a key component of the tissue healing process [54] or around the implants. Hyaluronic acid could be an excellent delivery system for stem cells and small molecules, such as our CH3-M6P, along with HA and TCP. In such a scaffold system, the gell will promote the proliferation and migration of cells to the injury site, while CH3-M6P induces angiogenesis and enhances the penetration of new microvessels through the porous structure of the scaffold. Therefore, our findings underscore the necessity of investigating CH3-M6P in bone scaffold fabrication, and its stabilization, highlighting its potential as a key component in future applications. We are thinking that, in the future, an adapted form of M6P can be checked with a combination of PRP together with stem cells. PRP is a combination of platelets, growth factors, and other bioactive molecules. Activated PRP has a higher concentration of growth factors than unactivated PRP. Various methods like calcium chloride activation and thrombin activation, and exogenous energy sources such as laser and ultrasound, have been reported for PRP activation up to today [55], and we are thinking that it is worth checking the CH3-M6P on PRP activation. Consequently, its effect on the speed of activation also has control over the degree of growth factors release, since, after the CH3-M6P treatment, we observed the induction of various genes on dental pulp stem cells. Our adapted chemical also can be checked along with PRP, since PRP is more affordable compared to stem cell therapies. With its lesser immune suppression, anti-apoptotic, anti-microbial, and anti-oxidant effects, and speculatively, we can say that, investigation of its aggregation properties (fast or slow) could be important to avoid leaking of injected material from the wound site (until the jelification in the local environment). This combined therapy could not just be good for bone regeneration, but also for tendon regeneration, cardiovascular diseases, hair loss, skin regeneration, sinus and ridge augmentation, and so on.

In conclusion, we demonstrated the M6P effect on mesenchymal stem cells, which could effectively solve the limitations caused by the lack of angiogenesis in clinical practice, as it alters the expression of some important osteogenic/angiogenic markers and microvessel outgrowth; however, our study still needs further progress due to experimental limitations such as protein expression in tissue.

Taking blood flow augmentation and shear stress into consideration further, we need to check its effect on the in vivo model, and in combination with HA/TCP, hyaluronic acid, and/or PRP. Synthesizing a panel of M6P derivatives and using liposomes is also a new perspective in order to cargo angiogenic/osteogenic agents into the stem cell via the M6P/IGF2 receptor, as liposomes can be decorated with M6P residues [56].

## Figures and Tables

**Table 1:** mRNA sequence for PCR analysis.

Gene	Sequence
Col 1	(F) AGAACTGGTACATCAGCAAG
	(R) GAGTTTACAGGAAGCAGACA
ALP	(F) CCAACGTGGCTAGAATGTCATC
	(R) TGGGCATTGGTGTTGTACGTC
RunX2	(F) TGGTTAATCTCCGCAGGTCAC
	(R) ACTGTGCTGAAGAGGCTGTTTG
Smad 3	(F) AAGTGCATCACCATCCCCAG
	(R) GGGTCAACTGGTAGACAGCC
TSP 1	(F) AAGACCTGGTGGATGCTGTG
	(R) GAAGACGCTTTGGATGGGGA
VEGF A	(F) CCATCCAATCGAGACCCTGG
	(R) TATGTGCTGGCCTTGGTGAG
Mef2C	(F) GCACCAACAAGCTGTTCCAG
	(R) TGTCTGAGTTTGTCCGGCTC
TGF-b1	(F) TGAACCGGCCTTTCCTGCTTCTCATG
	(R) GCGGAAGTCAATGTACAGCTGCCGC
TGF-b2	(F) GGCTCAACCACCAGGGCATCCAGAT
	(R) CTCCCCGAGAGCCTGTCCAGATGCT
GAPDH	(F) ATGGGGAAGATAAAGGTCG
	(R) TAAAAGCAGCCCTGGTGACC

## Data Availability

The original contributions presented in the study are included in the article, further inquiries can be directed to the corresponding authors.
